# Effects of music-based interventions on test anxiety in students: a systematic review and meta-analysis

**DOI:** 10.3389/fpsyg.2026.1922862

**Published:** 2026-08-20

**Authors:** Hongmin Zou, Chenyu Liu, Jiaxuan Luo, Ying Chen, Yupeng He, Yingcong Liu

**Affiliations:** 1 School of Educational Science, Quanzhou Normal University, Quanzhou, Fujian, China; 2 School of Music Education, Wuhan Conservatory of Music, Wuhan, Hubei, China

**Keywords:** meta-analysis, music-based interventions, students, systematic review, test anxiety

## Abstract

**Objective:**

Test anxiety is one of the most prevalent psychological problems among adolescents and university students and is associated with impaired academic performance, emotional distress, and reduced psychological wellbeing. This study aimed to systematically evaluate the effects of music-based interventions on test anxiety among students.

**Methods:**

A comprehensive literature search was conducted in CNKI, Wanfang Data, VIP Database, China Biology Medicine (CBM), PubMed, Web of Science, the Cochrane Library, and Embase from database inception to April 2026. Published randomized controlled trials (RCTs) investigating the effects of music-based interventions on test anxiety among students were included. Meta-analyses were performed using RevMan 5.4 and Stata 18.0. The primary outcomes were test anxiety measured using the State Anxiety Subscale of the State-Trait Anxiety Inventory (STAI-S/SAI), Test Anxiety Scale (TAS), Test Anxiety Inventory (TAI), and Generalized Test Anxiety Inventory (GTAI). Fixed- or random-effects models were applied according to heterogeneity, and subgroup analyses were conducted.

**Results:**

A total of 10 RCTs involving 681 students were included, with 371 participants in the intervention groups and 310 in the control groups. The meta-analysis showed that music-based interventions were significantly more effective than control conditions in reducing test anxiety among students (SMD = −1.21, 95% CI: −1.79 to −0.63, *P* < 0.0001). Subgroup analyses indicated that interventions lasting ≥8 weeks (SMD = −2.14), sessions lasting ≥60 min (SMD = −1.89), and active music-based interventions (SMD = −2.21) produced greater reductions in test anxiety.

**Conclusion:**

Music-based interventions appear to be a promising non-pharmacological strategy for improving students' psychological wellbeing by reducing test anxiety and may serve as an effective adjunct to school-based mental health education.

**Systematic review registration:**

https://www.crd.york.ac.uk/prospero/, identifier: CRD420261414547.

## 1 Introduction

Student mental health has become an important issue in global public health and education, with anxiety, depression, and stress being common among adolescents and university students (Pascoe et al., 2020). These psychological problems not only affect students' emotional wellbeing and social adjustment but are also closely associated with reduced academic engagement and poorer academic performance (Madigan and Curran, 2021). Among these problems, test anxiety, characterized by both situational anxiety and academic stress, has become one of the major challenges faced by students worldwide (Sapanci et al., 2026). Test anxiety is manifested not only as anxiety-related responses in examination settings, such as tension, worry, and physiological arousal (Živanović et al., 2024; Guo et al., 2024), but may also further impair students' cognitive functioning, learning efficiency, and examination performance (Santi et al., 2024). In severe cases, persistent test anxiety may even contribute to the development of anxiety and depressive disorders, thereby affecting students' mental health and overall development (Li et al., 2025; Raglio et al., 2026). In particular, students in primary and secondary schools as well as higher education are chronically exposed to academic competition, examination-based evaluation, and pressure for educational advancement; therefore, test anxiety may become an important factor affecting their psychological adjustment and academic development (Lu et al., 2024; Huang et al., 2025). Accordingly, exploring effective intervention strategies applicable to students at different educational stages is of great significance for promoting student mental health and improving the quality of school-based mental health services.

At present, interventions for test anxiety among students mainly include pharmacological treatment, psychological interventions, and behavioral interventions (Schaefer et al., 2019; Uysal et al., 2024). Pharmacological treatment may improve anxiety symptoms to some extent, but it is associated with concerns such as adverse effects, risk of dependence, and limitations in long-term use (Kumar et al., 2026; Pacnejer et al., 2025). Psychological and behavioral interventions, such as cognitive behavioral therapy, relaxation training, and systematic desensitization, have been shown to effectively reduce anxiety levels among students (Wimbarti et al., 2025). However, their effective implementation in school settings may be limited by students' access to these services and by practical implementation conditions. Music is highly accessible and feasible across diverse populations. As a psychological regulation approach based on musical stimulation or musical engagement, music-based intervention is considered to have potential benefits for alleviating anxiety, improving emotional states, and promoting mental health (Ugwuanyi et al., 2021). Accordingly, music-based interventions have attracted increasing attention in the fields of test anxiety and mental health (Gao and Liu, 2021).

The existing literature generally distinguishes two broad categories of music-related interventions: therapist-led, goal-oriented clinical music therapy and interventions primarily based on music listening (Pang, 2022). In the present study, “music-based interventions” were adopted as an operational term, and the key intervention characteristics of each included study were systematically recorded during data extraction to facilitate the interpretation of potential heterogeneity. Specifically, the operational definition used in this study refers to structured music activities designed to alleviate test anxiety and delivered according to a clearly defined intervention protocol, including music type, session duration, frequency, and overall intervention period. These interventions included active music-based interventions, such as instrumental performance, singing, improvisation, and recreative music activities, as well as receptive music-based interventions, such as listening to soothing music or natural sounds. Passive music playback intended solely for entertainment or background purposes was not included. Music used as an intervention can improve individuals' physiological, psychological, and emotional states (Shen et al., 2025; Dsouza et al., 2024). Evidence suggests that music may exert anxiolytic effects through mechanisms such as modulating autonomic nervous system activity, reducing cortisol levels, and diverting attention away from negative stimuli (Ramirez et al., 2025; Ubrangala et al., 2022). In recent years, music-based interventions have increasingly been applied to alleviate test anxiety among students (Parada-Cabaleiro et al., 2021), with several studies reporting beneficial effects (Liu et al., 2025; Song et al., 2024). However, existing studies vary substantially in intervention protocols, including music type, session duration, frequency, and overall intervention period, as well as in participants' educational stages and outcome measurement instruments. Therefore, evidence from high-quality systematic reviews and meta-analyses remains limited.

Although previous systematic reviews and meta-analyses have demonstrated the overall effects of music-based interventions on anxiety from different perspectives, the evidence base for test anxiety, as a context-specific form of anxiety, remains substantially limited.

de Witte et al. (2025) conducted a multilevel meta-analysis of anxiety. Including 51 studies and 93 effect sizes, the study demonstrated that music therapy had a significant moderate effect on anxiety (*g* = 0.357), with receptive and combined approaches yielding significantly larger effects than active-only approaches (de Witte et al., 2025). Although the study covered both state and trait anxiety, it did not examine test anxiety as a separate outcome in subgroup analyses. Moreover, only a limited proportion of the included primary studies involved examination-related contexts; therefore, the direct applicability of these findings to test anxiety remains unclear.

Harney et al. (2023) conducted a systematic review of naturally occurring state anxiety, including 24 controlled studies, and reported a large beneficial effect of music listening (*d* = −0.77) (Harney et al., 2023). Although student populations were included, state anxiety across multiple contexts was pooled, and test anxiety was not evaluated separately. In addition, because experimentally induced anxiety was excluded and the review focused on naturally occurring anxiety, its effect size cannot directly reflect the specific therapeutic effects of music listening in the examination-related stress context.

Li et al. (2026) conducted a meta-analysis of anxiety among college students, including 18 RCTs and 1,679 participants, and found that music therapy significantly reduced anxiety levels (SMD = −1.54) (Li et al., 2026). However, the inclusion criteria were relatively broad, covering multiple types of anxiety, including social anxiety, generalized anxiety, test anxiety, employment-related anxiety, and performance anxiety, without subgroup analyses by anxiety subtype. Therefore, the differential effects of music-based interventions on different anxiety types could not be distinguished. Notably, although test anxiety was mentioned in the introduction as one of the common anxiety types among students, the pooled effect size represented the overall effect of music-based interventions on “college student anxiety,” rather than an efficacy evaluation specifically targeting examination-related contexts.

Chen et al. (2026) conducted a network meta-analysis including 28 RCTs and 2,654 participants to compare the relative efficacy of eight types of art-based interventions. The results showed that cognitive behavioral therapy combined with music therapy ranked highest for anxiety symptoms (SUCRA = 92.4%), whereas music therapy alone ranked first for depressive symptoms (SUCRA = 82.4%) (Chen et al., 2026). However, that study did not separately analyze test anxiety, and its outcome indicators focused on generalized anxiety symptoms, failing to clarify the applicability of music-based interventions in the specific stress context of examinations.

Taken together, although these four high-quality meta-analyses have demonstrated the efficacy of music-based interventions for anxiety from different perspectives, their shared limitation is that test anxiety was addressed only as one subtype or dimension of anxiety, without an independent and systematic evaluation of the context-specific effects of music-based interventions on test anxiety. In addition, although the meta-analysis by Li et al. (2026) was the most recently published, only a limited number of its included primary studies explicitly focused on test anxiety, and it did not conduct systematic subgroup analyses according to intervention duration, session duration, intervention modality, or target population to explore optimal intervention parameters.

Based on the above analysis, the necessity of the present study is reflected in the following aspects: (1) existing reviews have not specifically focused on the question of “music-based interventions for test anxiety among students.” As a context-specific form of anxiety in academic settings, test anxiety may differ from general anxiety in its eliciting mechanisms, maintenance factors, and responses to intervention; (2) several recently published randomized controlled trials on test anxiety from 2024 to 2026 have not been included in previous meta-analyses, indicating that the evidence requires updating; and (3) secondary school students and university students are exposed to different examination-related stress contexts, and their responses to intervention may differ systematically. However, no study has directly compared these differences through subgroup analyses.

The present study makes several clear contributions to the existing evidence base: (1) it is the first to specifically focus on the question of “music-based interventions for test anxiety among students,” including 10 RCTs, several of which were published after 2024, and provides an independent evaluation of intervention effects in examination-related contexts; (2) it is the first to systematically conduct multidimensional subgroup analyses according to intervention duration, session duration, intervention modality, and target population, thereby helping to identify optimal intervention parameters and reveal differential effects across intervention formats; (3) it is the first meta-analysis to distinguish the effects of active music therapy from receptive music listening in alleviating test anxiety; and (4) it is the first to compare effect size differences between secondary school students and university students receiving music-based interventions for test anxiety, providing evidence to inform stratified intervention strategies in school-based mental health services.

The present study aimed to systematically evaluate the effects of music-based interventions on test anxiety among students through meta-analysis and to synthesize the latest evidence available up to April 2026. Specifically, this study sought to determine the efficacy of music-based interventions for reducing test anxiety among students and to explore key factors that may influence intervention effects, including intervention duration, session duration, intervention modality, and target population. The findings are expected to provide evidence-based support for integrating music-based interventions into school-based mental health service systems.

## 2 Methods

### 2.1 Registration and protocol

This systematic review and meta-analysis was registered in the International Prospective Register of Systematic Reviews (PROSPERO; registration no. CRD420261414547). The study was conducted according to a pre-defined protocol and reported in accordance with the Preferred Reporting Items for Systematic Reviews and Meta-Analyses (PRISMA) guidelines.

### 2.2 Search strategy

A comprehensive literature search was conducted in CNKI, Wanfang Data, VIP Database, China Biology Medicine Database (CBM), PubMed, Web of Science, the Cochrane Library, and Embase. The search period covered each database from inception to April 2026. Chinese search terms included “音乐治疗(music therapy),” “音乐干预(music intervention),” “音乐疗法(music treatment),” “五音疗法(five-tone therapy),” “考试焦虑(test anxiety),” “测验焦虑(test anxiety),” “学业焦虑(academic anxiety),” “考前焦虑(pre-examination anxiety),” “学生(student),” “大学生(college/university student),” “中学生(secondary school student),” “高中生(high school student),” “初中生(junior high school student),” and “小学生(primary school student).” English search terms included “music,” “music therapy,” “music intervention,” “test anxiety,” “exam anxiety,” “academic anxiety,” “student,” “college student,” “university student,” and “high school student.” Searches combined subject headings with free-text terms. Only studies published in Chinese or English were included. The complete search strategies for each database are provided in the Supplementary material.

### 2.3 Inclusion and exclusion criteria

Inclusion criteria: studies were included if they met the following criteria: (1) participants: students enrolled in schools or other educational institutions who were identified as having test anxiety using standardized scales, such as the TAS, TAI, STAI-S, or GTAI, with no restrictions on sex or grade level; (2) interventions: participants in the experimental groups received music-based interventions, including active approaches such as playing musical instruments or singing, or receptive approaches such as listening to music; (3) comparators: control groups received no intervention, relaxation training, or other non-music interventions; (4) study design: randomized controlled trials (RCTs); (5) language: studies published in Chinese or English.

Exclusion criteria: studies were excluded if they met any of the following criteria: (1) participants were not students, or were students formally diagnosed by clinicians with an anxiety disorder according to DSM-5 or ICD-11 criteria; (2) the intervention was not music-centered, for example, music was used only as background sound, or music-based interventions were combined with other non-music interventions, such as cognitive behavioral therapy, exercise interventions, or mindfulness meditation, making it impossible to independently evaluate the effect of the music-based intervention; (3) full texts were unavailable or outcome data were incomplete; (4) the study used a non-RCT design, such as a cross-sectional study, case-control study, cohort study, or quasi-experimental design; (5) the publication was not an original research article, such as a conference paper, dissertation, review, systematic review, meta-analysis, animal study, or case report; (6) the study was judged as having a high risk of bias using the Cochrane Risk of Bias Tool, with serious bias in multiple key domains that could compromise the credibility of the findings; (7) the study was not published in Chinese or English.

Outcome measures: this study did not pre-specify a single specific scale; instead, it accepted all standardized instruments that assessed anxiety states in examination-related contexts as eligible outcome measures. These included the State Anxiety Subscale of the State-Trait Anxiety Inventory (STAI-S/SAI), when used to assess anxiety states in examination-related situations; scales specifically designed to measure test anxiety, such as the TAS and TAI; and the Generalized Test Anxiety Inventory (GTAI), which is applicable to secondary school students. The outcome measures were required to directly assess the concept of “test/exam anxiety,” namely students' tension, worry, and physiological arousal responses in examination or similar evaluative situations.

Outcomes were treated as continuous variables, and included studies were required to report either pre- and post-intervention scale scores or between-group comparisons of post-intervention scores. If an included study used another standardized test anxiety scale not listed above, the reliability and validity of that scale had to be clearly reported in the article, and the scale was included in the analysis only after two reviewers confirmed that its measurement target was test anxiety. Outcome types were excluded if they involved general anxiety scales that did not explicitly assess “test anxiety” or specify an examination-related context, such as scales reporting only “anxiety”; self-developed scales without reliability and validity validation; or studies reporting only physiological indicators, such as heart rate, blood pressure, or cortisol, without subjective anxiety scores.

Because different studies may have used different scales to measure the same construct, namely test anxiety, standardized mean differences (SMDs) were used as the pooled effect size in the meta-analysis. The SMD converts scores from different measurement scales into a common effect metric, thereby allowing pooled analyses across studies. This approach is recommended by the Cochrane Handbook and represents a standard statistical strategy for synthesizing the same type of outcome measured using different instruments.

### 2.4 Data extraction

All retrieved records were imported into EndNote 21 reference management software (McKeown and Mir, 2021), and duplicate records were identified and removed using its automatic deduplication function. Subsequently, two reviewers manually checked the remaining records by comparing titles, authors, and journal names item by item to ensure complete removal of duplicates. After this two-step deduplication process, two reviewers independently screened the titles and abstracts of the remaining records according to the pre-defined inclusion and exclusion criteria to exclude studies that were clearly ineligible. For studies that were uncertain or preliminarily eligible, the full texts were further reviewed to determine final eligibility.

Two reviewers independently extracted data using a pre-designed Excel data extraction form (Microsoft Excel 2021) (Winston, 2021). The extracted information included the first author, year of publication, country, sample size, participant characteristics (age and sex), intervention details (music type, intervention modality, session duration, frequency, and overall intervention period), control conditions, outcome measures (measurement instruments and score data), and key intervention parameters. After data extraction was completed, the two reviewers cross-checked the extracted results item by item.

During the study screening and data extraction processes, any disagreements between the two reviewers regarding study eligibility or extracted data were resolved through discussion and consensus. If disagreements persisted, a third reviewer examined the original article and made the final decision to ensure the accuracy of study selection and the consistency of data extraction. The entire process of study selection and data extraction strictly followed the methodological recommendations of the Cochrane Handbook for Systematic Reviews (Higgins et al., 2019) and was reported in accordance with the PRISMA 2020 statement (Page et al., 2021).

To comprehensively describe the heterogeneity characteristics of the included studies and provide a basis for qualitative analysis, two reviewers also extracted information on intervention delivery modes and intervention settings. Intervention delivery modes were classified as active music-based interventions, in which participants engaged through instrumental performance, singing, improvisation, or similar activities, and receptive music-based interventions, in which participants primarily listened to music. Intervention settings were categorized according to the descriptions in the original articles, including activity rooms, classrooms, simulation laboratories, and mental health education centers. In addition, information on music type, such as classical music, folk music, or light music, intervention frequency, session duration, overall intervention period, and outcome assessment time points, including pre-test, post-test, and follow-up, was extracted.

**Figure 1:**
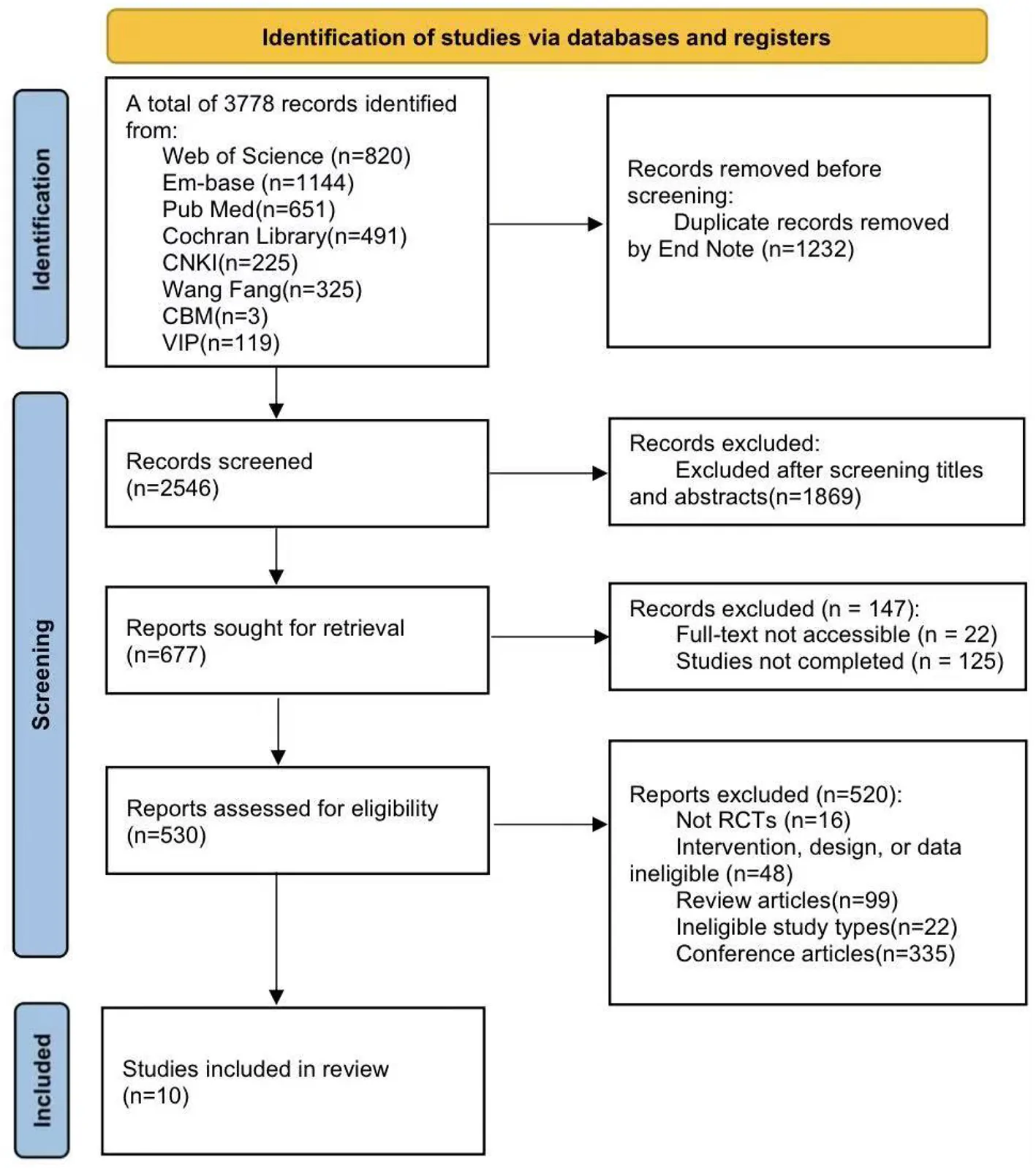
PRISMA flow diagram of study selection.

The above characteristics of the included studies were presented in a structured (Table 1) to provide a clear overview of heterogeneity across studies in terms of populations, intervention methods, and outcome measurements. The study screening process and reasons for exclusion were visually presented using a PRISMA flow diagram (Figure 1). The extraction and presentation of this qualitative information provided a basis for understanding between-study heterogeneity and interpreting the subsequent meta-analysis results.

**Table 1:** Characteristics of the included studies.

Source	Country	Participants	Group size/age		N		Intervention description		Intervention setting	Outcome measurement timepoints	Musical features	Frequency (sessions/ week)	Session length (min)	Duration (weeks)	Outcomes
			IG	CG	IG	CG	IG	CG							
Sagirkaya and Torun, 2026	Turkey	High school seniors	N/S	N/S	26	23	AMI	Routine learning	Activity room	Pre-test, post-test	N/S	1	≥60	≥8	TAS
Ugwuanyi et al., 2020	Nigeria	High school science seniors	18.98 ± 5.45	18.23 ± 4.67	46	37	AMI	No intervention	N/S	Pre-test, post-test, follow-up	Multiple genres	1	≥60	≥8	GTAI
Inangil et al., 2020	Turkey	Undergraduate nursing students	18.9 ± 0.88	19.5 ± 1.07	30	30	PMI	No intervention	Activity room	Pre-test, post-test	Traditional Turkish music	1	< 30	< 4	SAI
Ergin et al., 2025	Turkey	Second-year undergraduate nursing students	20.48 ± 1.24	20.41 ± 1.04	40	37	PMI	Routine simulation preparation	Activity room	Pre-test, post-intervention, post-simulation	Western classical music	1	30- < 60	< 4	SAI
Contreras, 2022	USA	First-year undergraduate nursing students	27.55 ± 6.63	24.75 ± 4.47	49	40	PMI	No intervention	Simulation laboratory	Pre-test, post-intervention, post-skill assessment	Classical crossover	1	< 30	< 4	SAI
Liang, 2024	China	Undergraduate students	N/S	N/S	15	15	AMI	No intervention	N/S	Pre-test, post-test, follow-up	Light music	2	N/S	4- < 8	TAI
Song et al., 2024	China	Undergraduate medical students	19.72 ± 1.07	19.83 ± 1.05	29	30	AMI	No intervention	Mental health education center	Pre-test, post-test, follow-up	Improvised creation	1	≥60	4- < 8	TAS
Song et al., 2025	China	Undergraduate medical students	20.85 ± 1.02	19.28 ± 0.70	32	29	PMI	Neutral stimulus	Activity room	Pre-test, post-test	Chinese folk music	1	< 30	< 4	SAI
Qin et al., 2012	China	Undergraduate medical students	19.2 ± 0.8	19.4 ± 1.0	90	55	PMI	No intervention	Activity room	Pre-test, post-test	Western classical music	2	30- < 60	4- < 8	TAI
Yang, 2015	China	Third-year junior high school students	N/S	N/S	14	14	PMI	Routine learning	Activity room	Pre-test, Post-test	Popular music, piano pieces, etc.	1	≥60	≥8	SAI

IG, intervention group; CG, control group; N/S, not specified; AMI, active music intervention; PMI, passive music intervention; TAS, Test Anxiety Scale; GTAI, Generalized Test Anxiety Inventory; SAI, State Anxiety Inventory; TAI, Test Anxiety Inventory.

### 2.5 Quality assessment

The above characteristics of the included studies were presented in a structured (Table 1) to provide a clear overview of heterogeneity across studies in terms of populations, intervention methods, and outcome measurements. The study screening process and reasons for exclusion were visually presented using a PRISMA flow diagram (Figure 1). The extraction and presentation of this qualitative information provided a basis for understanding between-study heterogeneity and interpreting the subsequent meta-analysis results.

### 2.6 Data synthesis and analysis

Meta-analyses were performed using RevMan 5.4 (Tantry et al., 2021) and Stata 18.0 (Unguryanu and Grjibovski, 2014). All outcomes included in this study were continuous variables. Because the test anxiety scales used across studies were not identical, including the STAI-S, TAS, TAI, and GTAI, standardized mean differences (SMDs) with corresponding 95% confidence intervals (CIs) were used as the pooled effect estimates to account for differences in scoring scales across instruments. All pooled analyses were weighted using the inverse-variance method.

Statistical heterogeneity was assessed using Cochran's *Q*-test and the *I*^2^ statistic. If *I*^2^ < 50% and *P* > 0.10, between-study heterogeneity was considered low, and a fixed-effect model was applied. If *I*^2^ ≥ 50% or *P* ≤ 0.10, substantial heterogeneity was considered to be present, and a random-effects model was used, with τ^2^ estimated using the DerSimonian-Laird method.

Sensitivity analysis was performed using a leave-one-out approach. Specifically, each study was sequentially excluded, and the pooled effect size was recalculated to examine the range of changes in the pooled estimate and whether statistical significance changed, thereby assessing the robustness of the results.

When the number of included studies was ≥10, funnel plots were generated for visual assessment, and Egger's regression test was used to quantitatively evaluate publication bias, with the significance level set at *P* < 0.05. When the number of included studies was limited (< 10) or heterogeneity was substantial, the statistical power of funnel plots and Egger's test may be insufficient; therefore, the corresponding results were considered exploratory and interpreted with caution.

## 3 Results

### 3.1 Study selection

During the full-text review stage, a total of 520 records were excluded for the following reasons: non-randomized controlled trials (*n* = 16); interventions, study designs, or outcome data that did not meet the inclusion criteria (*n* = 48); review articles (*n* = 99); ineligible publication types, including letters to the editor, brief commentaries, pre-prints, survey reports, empty records with bibliographic information only and no full-text content, and studies published in languages other than Chinese or English (*n* = 22); and conference abstracts or conference papers (*n* = 335).

After the above screening process, 10 studies published in Chinese or English were finally included, involving a total of 681 students, with 371 participants in the intervention groups and 310 in the control groups. The study screening and selection process is presented in Figure 1.

### 3.2 Characteristics of the included studies

The main characteristics of the included studies are presented in Table 1. The 10 included studies were published between 2012 and 2026 and were conducted in Türkiye, China, the United States, and Nigeria. All studies used randomized controlled trial or quasi-experimental designs.

Participants included high school students, junior high school students, undergraduates, medical students, and nursing students, with sample sizes ranging from 30 to 145.

The interventions were classified into two categories: active and receptive modalities. Four studies (Sagirkaya and Torun, 2026; Ugwuanyi et al., 2020; Liang, 2024; Song et al., 2024) used active music-based interventions, including improvisation, songwriting, recreative music activities, and music-based cognitive behavioral therapy, with intervention periods ranging from 4 to 12 weeks and session durations ranging from 60 to 90 min. Six studies (Inangil et al., 2020; Ergin et al., 2025; Contreras, 2022; Song et al., 2025; Qin et al., 2012; Yang, 2015) used receptive music-based interventions based on music listening, with session durations ranging from 7 to 30 min; these were mostly single-session or short-term interventions. Intervention settings mainly included school activity rooms, classrooms, and mental health education centers.

Regarding outcome assessment time points, all 10 studies (Sagirkaya and Torun, 2026; Ugwuanyi et al., 2020; Inangil et al., 2020; Ergin et al., 2025; Contreras, 2022; Liang, 2024; Song et al., 2024, 2025; Qin et al., 2012; Yang, 2015) reported pre- and post-intervention data. Three studies (Ugwuanyi et al., 2020; Liang, 2024; Song et al., 2024) additionally conducted follow-up assessments ranging from 2 weeks to 2 months, and two studies (Ergin et al., 2025; Contreras, 2022) measured outcomes both immediately after the intervention and after skill-based examinations.

Music characteristics varied substantially across studies. Three studies (Ergin et al., 2025; Contreras, 2022; Qin et al., 2012) used Western classical music, two studies (Inangil et al., 2020; Song et al., 2025) used culturally specific music, including Chinese folk music and Turkish traditional music, one study (Liang, 2024) used light music, one study (Ugwuanyi et al., 2020) used multiple types of music, one study (Yang, 2015) selected pop music based on students' preferences, one study (Song et al., 2024) involved improvisation without fixed musical pieces, and one study (Sagirkaya and Torun, 2026) did not clearly report the music type.

### 3.3 Risk of bias assessment

**Figure 2:**
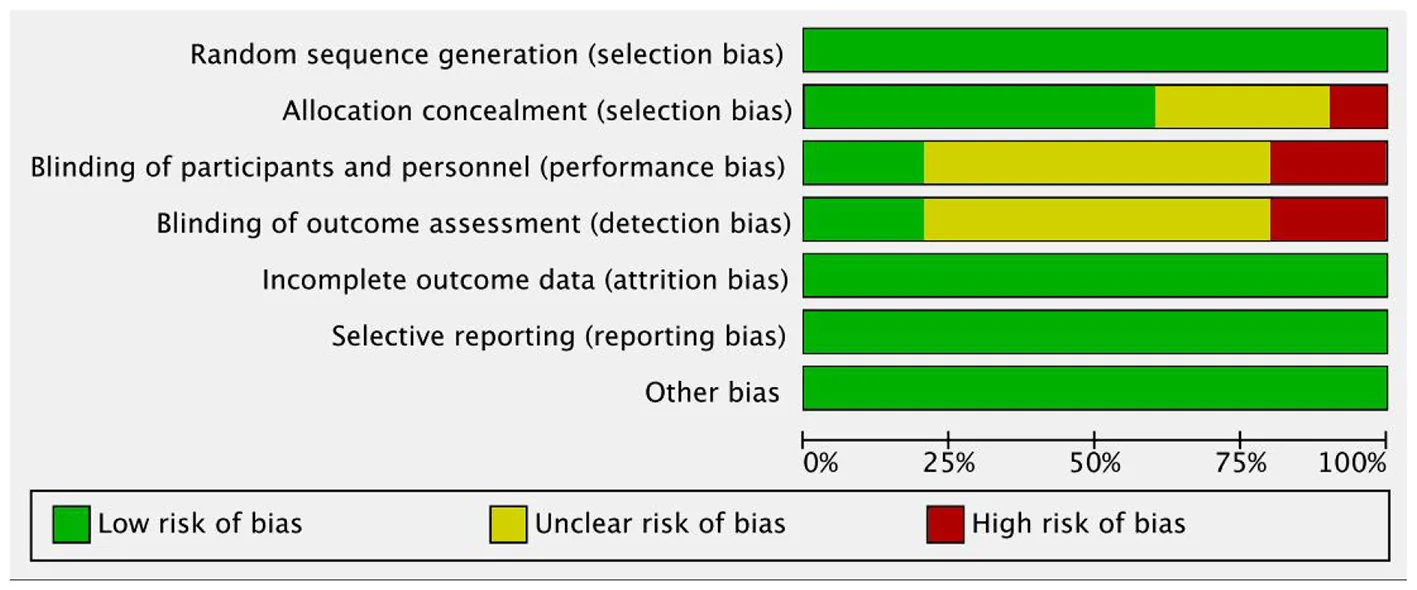
Risk of bias summary of the included studies.

**Figure 3:**
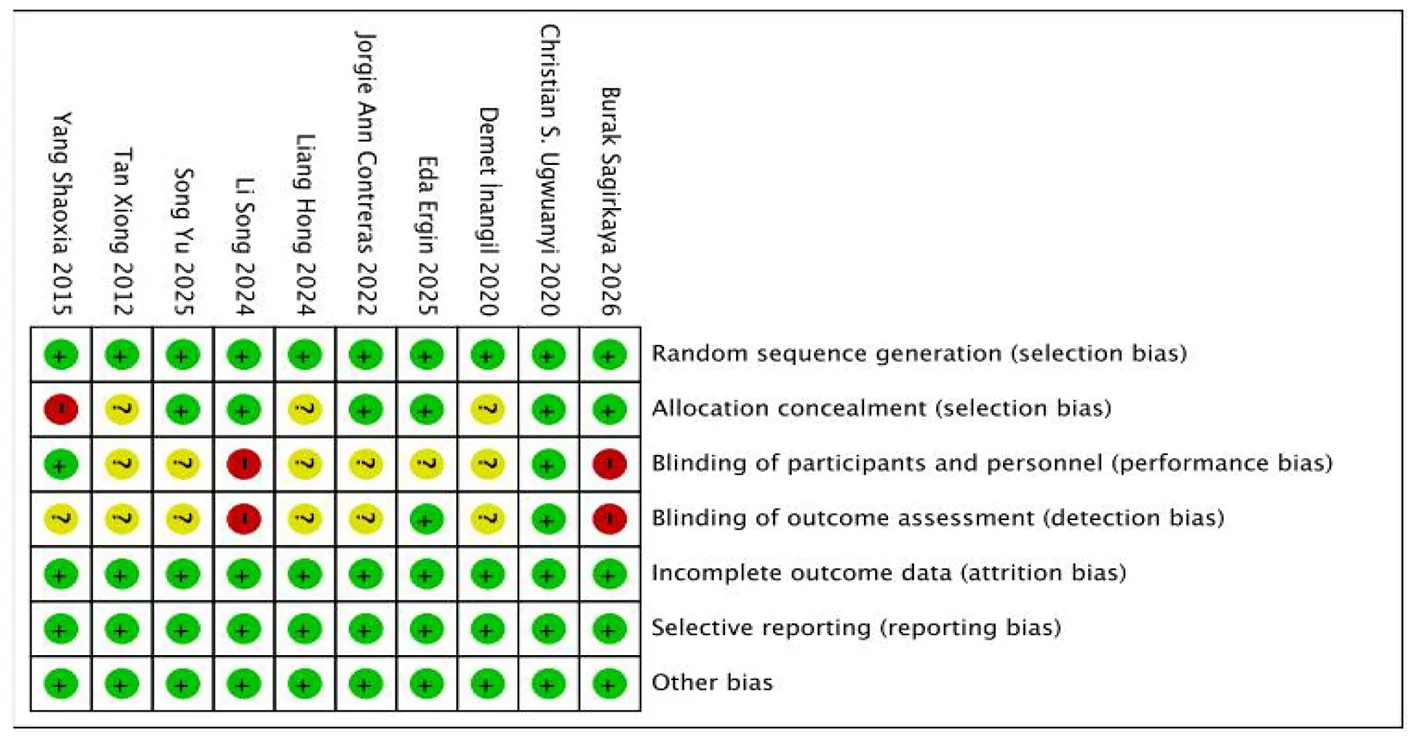
Risk of bias graph of the included studies.

The methodological quality of the 10 included studies was assessed using the Cochrane Risk of Bias Tool, and the results are presented in Figures 2, 3. For random sequence generation, all 10 studies were judged to have a low risk of bias. Regarding allocation concealment, six studies (Sagirkaya and Torun, 2026; Ugwuanyi et al., 2020; Ergin et al., 2025; Contreras, 2022; Song et al., 2024, 2025) reported specific randomization procedures, including sealed opaque envelopes, random number tables, and computer-generated random allocation. Because of the inherent nature of music-based interventions, blinding both participants and intervention providers was challenging. Only two studies (Ugwuanyi et al., 2020; Yang, 2015) reported blinding of participants and personnel, and two studies (Ugwuanyi et al., 2020; Ergin et al., 2025) reported blinding of outcome assessors. Notably, two studies (Sagirkaya and Torun, 2026; Song et al., 2024) were judged to have a high risk of bias in both blinding domains, whereas the remaining studies did not report relevant blinding procedures. Nevertheless, no substantial attrition, missing outcome data, or other potential sources of bias were identified. Therefore, all studies were judged to have a low risk of bias for incomplete outcome data, selective outcome reporting, and other potential sources of bias.

### 3.4 Meta-analysis

All 10 included studies reported the effects of music-based interventions on test anxiety or state anxiety among students, involving a total of 681 participants. Because different anxiety scales were used across studies, including the TAS, GTAI, SAI, and TAI, standardized mean differences (SMDs) were calculated for the pooled analysis. Substantial heterogeneity was observed among the studies (*I*^2^ = 91%, *P* < 0.00001); therefore, a random-effects model was applied.

**Figure 4:**
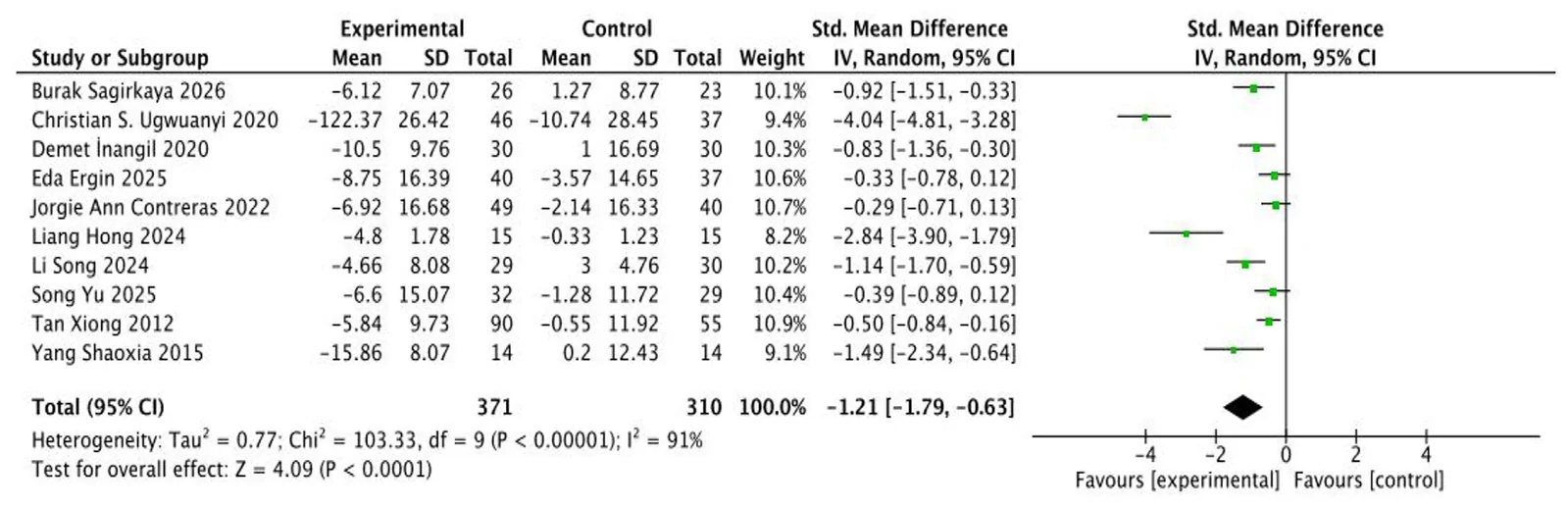
Forest plot of the effects of music-based interventions on test anxiety.

The pooled analysis showed that students receiving music-based interventions had significantly lower test anxiety scores than those in the control groups (SMD = −1.21, 95% CI: −1.79 to −0.63, *P* < 0.00001; Figure 4). These findings indicate that music-based interventions effectively reduce test anxiety among students, with a moderate-to-large effect size.

**Figure 5:**
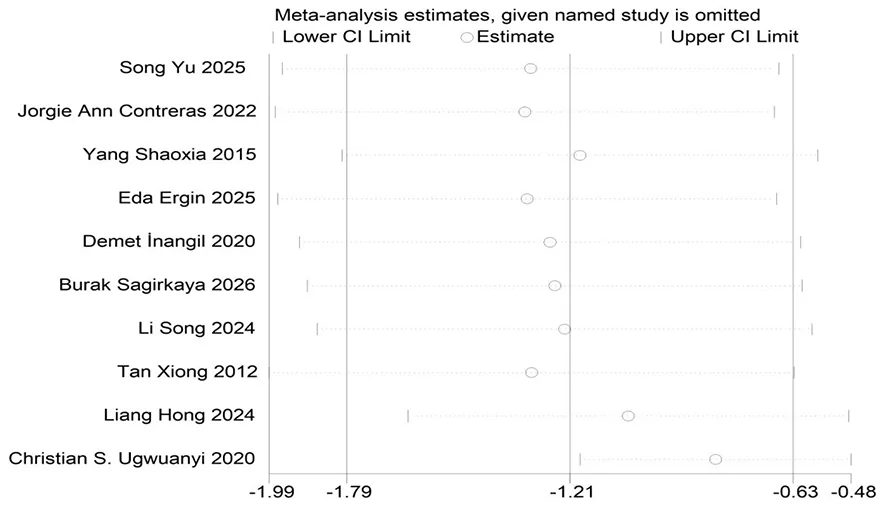
Sensitivity analysis of the effects of music-based interventions on test anxiety.

A leave-one-out sensitivity analysis was conducted to assess the robustness of the pooled effect estimate. After sequentially excluding each study, the pooled SMD ranged from −1.79 to −0.63, with a consistent direction of effect and statistical significance across all analyses. These findings indicate that the results were robust and that no individual study exerted a decisive influence on the pooled effect estimate (Figure 5).

**Figure 6:**
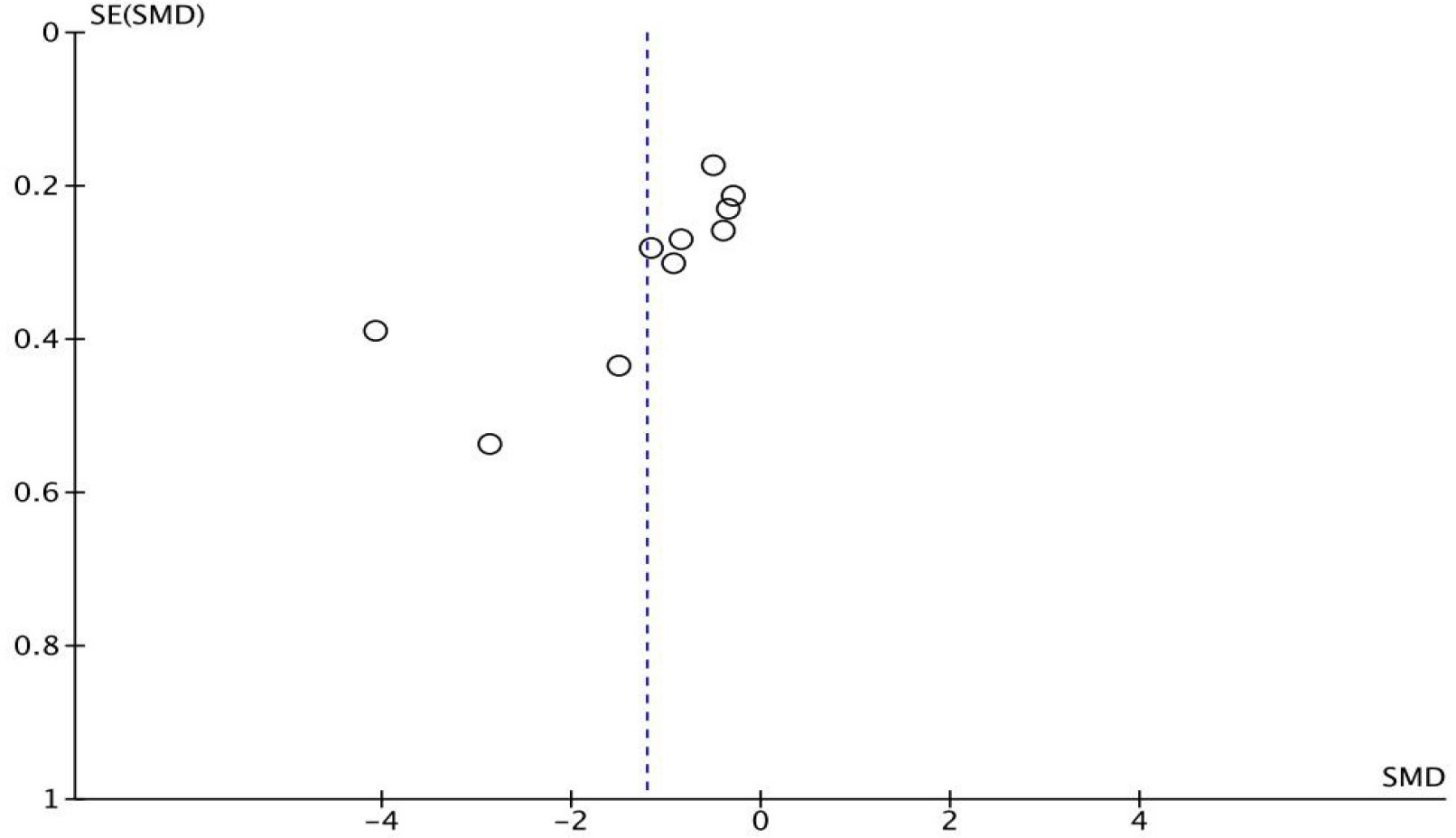
Funnel plot of the effects of music-based interventions on test anxiety.

As 10 studies were included, meeting the minimum requirement for funnel plot analysis, a funnel plot was generated to assess potential publication bias. The SMD of each study was plotted against its standard error (Figure 6). The funnel plot showed some asymmetry, suggesting possible publication bias or small-study effects. This may be attributable to the non-publication of studies with negative findings or the tendency of small studies to report larger effect estimates.

To further assess and adjust for potential publication bias, Egger's linear regression test and the trim-and-fill method were applied. Egger's test indicated statistically significant publication bias (bias = −8.26, *P* = 0.011). After adjustment using the trim-and-fill method, the pooled SMD under the random-effects model was −1.468 (95% CI: −2.112 to −0.824, *P* < 0.001). The direction and statistical significance of the effect remained unchanged compared with the original analysis, suggesting that publication bias had a limited influence on the overall conclusions and that the findings remained robust.

### 3.5 Subgroup analysis

To explore potential sources of between-study heterogeneity and further identify the optimal music-based intervention protocol, subgroup analyses were conducted according to overall intervention duration, session duration, intervention frequency, intervention modality, and participants' educational stage (Table 2).

**Table 2:** Subgroup analyses of the effects of music-based interventions on test anxiety.

Subgroups	No. of studies	Total sample (*I* + C)	Heterogeneity		MD (95% CI)	Meta-analysis results	
			*I* (%)	*P*-value		Z	*P*
Duration (weeks)							
< 4	4	287	0	0.41	−0.43 [−0.66, −0.19]	3.56	0.0004
4- < 8	3	234	89	< 0.0001	−1.37 [−2.42, −0.33]	2.59	0.01
≥8	3	160	95	< 0.00001	−2.14 [−4.07, −0.22]	2.19	0.03
Session length (minutes)							
< 30	3	210	24	0.27	−0.46 [−0.74, −0.19]	3.30	0.0010
30- < 60	2	222	0	0.56	−0.44 [−0.71, −0.16]	3.14	0.002
≥60	4	219	94	< 0.00001	−1.89 [−3.22, −0.55]	2.77	0.006
Frequency (sessions/week)							
2	2	175	94	< 0.0001	−1.61 [−3.91, 0.68]	1.38	0.17
1	8	506	92	< 0.00001	−1.14 [−1.82, −0.46]	3.28	0.001
Type of intervention							
Passive music intervention	6	460	40	0.14	−0.50 [−0.69, −0.31]	5.20	< 0.00001
Active music intervention	4	221	94	< 0.00001	−2.21 [−3.65, −0.77]	3.00	0.003
Target population							
Secondary school students	3	160	95	< 0.00001	−2.14 [−4.07, −0.22]	2.19	0.03
College students	7	521	77	0.0002	−0.75 [−1.15, −0.36]	3.72	0.0002

#### 3.5.1 Subgroup analysis by intervention duration

The effect size of music-based interventions on test anxiety increased with longer intervention duration, with long-term, repeated interventions producing greater effects than single-session interventions. To examine this factor, the included studies were divided into three subgroups: < 4 weeks, 4 to < 8 weeks, and ≥8 weeks. The < 4-week subgroup included four studies (Inangil et al., 2020; Ergin et al., 2025; Contreras, 2022; Song et al., 2025) and showed low heterogeneity (*I*^2^ = 0%, *P* = 0.41); therefore, a fixed-effect model was applied. The 4 to < 8-week subgroup, including three studies (Liang, 2024; Song et al., 2024; Qin et al., 2012), showed substantial heterogeneity (*I*^2^ = 89%, *P* < 0.0001), and the ≥8-week subgroup, including three studies (Sagirkaya and Torun, 2026; Ugwuanyi et al., 2020; Yang, 2015), also showed substantial heterogeneity (*I*^2^ = 95%, *P* < 0.00001); therefore, random-effects models were applied. The meta-analysis showed that music-based interventions significantly reduced test anxiety in all three subgroups, with pooled effect estimates of SMD = −0.43 (95% CI: −0.66 to −0.19, *P* = 0.0004), SMD = −1.37 (95% CI: −2.42 to −0.33, *P* = 0.01), and SMD = −2.14 (95% CI: −4.07 to −0.22, *P* = 0.03), respectively. The pooled effect size increased with longer intervention duration, suggesting that repeated interventions lasting ≥4 weeks may be more effective than interventions lasting < 4 weeks, with longer intervention periods producing greater benefits.

#### 3.5.2 Subgroup analysis by session duration

Session duration was an important factor influencing intervention effectiveness, with sessions lasting ≥60 min producing a substantially larger pooled effect than shorter sessions. To examine this factor, the included studies were divided into three subgroups: < 30 min, 30 to < 60 min, and ≥60 min. The < 30-min subgroup, including three studies (Inangil et al., 2020; Contreras, 2022; Song et al., 2025), showed low heterogeneity (*I*^2^ = 24%, *P* = 0.27), and the 30 to < 60-min subgroup, including two studies (Ergin et al., 2025; Qin et al., 2012), also showed low heterogeneity (*I*^2^ = 0%, *P* = 0.56); therefore, fixed-effect models were applied. The ≥60-min subgroup, including four studies (Sagirkaya and Torun, 2026; Ugwuanyi et al., 2020; Song et al., 2024; Yang, 2015), showed substantial heterogeneity (*I*^2^ = 94%, *P* < 0.00001); therefore, a random-effects model was applied. Music-based interventions significantly reduced test anxiety in all three subgroups, with pooled effect estimates of SMD = −0.46 (95% CI: −0.74 to −0.19, *P* = 0.001), SMD = −0.44 (95% CI: −0.71 to −0.16, *P* = 0.002), and SMD = −1.89 (95% CI: −3.22 to −0.55, *P* = 0.006), respectively. The effect was markedly greater in the ≥60-min subgroup than in the shorter-duration subgroups, suggesting that sessions lasting ≥60 min may produce greater benefits.

#### 3.5.3 Subgroup analysis by intervention frequency

Current evidence indicates that one music-based intervention session per week may be sufficient to significantly reduce test anxiety, whereas the additional benefits of higher intervention frequency require further investigation. The included studies were divided into once-weekly and twice-weekly subgroups. The once-weekly subgroup, including eight studies (Sagirkaya and Torun, 2026; Ugwuanyi et al., 2020; Inangil et al., 2020; Ergin et al., 2025; Contreras, 2022; Song et al., 2024, 2025; Yang, 2015), showed substantial heterogeneity (*I*^2^ = 92%, *P* < 0.00001), and the twice-weekly subgroup, including two studies (Liang, 2024; Qin et al., 2012), also showed substantial heterogeneity (*I*^2^ = 94%, *P* < 0.0001); therefore, random-effects models were applied for both subgroups. The pooled effect was statistically significant in the once-weekly subgroup (SMD = −1.14, 95% CI: −1.82 to −0.46, *P* = 0.001), whereas the pooled effect in the twice-weekly subgroup was not statistically significant (SMD = −1.61, 95% CI: −3.91 to 0.68, *P* = 0.17). These findings suggest that one session per week may be sufficient to produce a significant effect. Although the twice-weekly subgroup had a larger point estimate, the result remains uncertain because of the limited number of included studies and substantial heterogeneity.

#### 3.5.4 Subgroup analysis by intervention modality

Active music-based interventions produced greater reductions in test anxiety than receptive music-based interventions. To compare the two modalities, the included studies were divided into receptive and active music-based intervention subgroups. The receptive music-based intervention subgroup, including six studies (Inangil et al., 2020; Ergin et al., 2025; Contreras, 2022; Song et al., 2025; Qin et al., 2012; Yang, 2015), showed relatively low heterogeneity (*I*^2^ = 40%, *P* = 0.14); therefore, a fixed-effect model was applied. The active music-based intervention subgroup, including four studies (Sagirkaya and Torun, 2026; Ugwuanyi et al., 2020; Liang, 2024; Song et al., 2024), showed substantial heterogeneity (*I*^2^ = 94%, *P* < 0.00001); therefore, a random-effects model was applied. Significant reductions in test anxiety were observed in both the receptive music-based intervention subgroup (SMD = −0.50, 95% CI: −0.69 to −0.31, *P* < 0.00001) and the active music-based intervention subgroup (SMD = −2.21, 95% CI: −3.65 to −0.77, *P* = 0.003). The larger pooled effect in the active music-based intervention subgroup suggests that activities such as musical improvisation and group music creation may be more effective than receptive music-based interventions.

#### 3.5.5 Subgroup analysis by educational stage

Music-based interventions were effective across different educational stages, with a larger effect observed among secondary school students. To compare intervention effects across educational stages, the included studies were divided into secondary school and university student subgroups. The secondary school subgroup, including three studies (Sagirkaya and Torun, 2026; Ugwuanyi et al., 2020; Yang, 2015), showed substantial heterogeneity (*I*^2^ = 95%, *P* < 0.00001), and the university student subgroup, including seven studies (Inangil et al., 2020; Ergin et al., 2025; Contreras, 2022; Liang, 2024; Song et al., 2024, 2025; Qin et al., 2012), also showed substantial heterogeneity (*I*^2^ = 77%, *P* = 0.0002); therefore, random-effects models were applied for both subgroups. Music-based interventions significantly reduced test anxiety in both secondary school students (SMD = −2.14, 95% CI: −4.07 to −0.22, *P* = 0.03) and university students (SMD = −0.75, 95% CI: −1.15 to −0.36, *P* = 0.0002). The larger effect observed among secondary school students may reflect their greater academic pressure and higher baseline levels of test anxiety; however, it may also have been influenced by the limited number of included studies and substantial heterogeneity.

## 4 Discussion

### 4.1 Effects of music-based interventions on students' test anxiety and potential mechanisms

This study included 10 randomized controlled trials (Sagirkaya and Torun, 2026; Ugwuanyi et al., 2020; Inangil et al., 2020; Ergin et al., 2025; Contreras, 2022; Liang, 2024; Song et al., 2024, 2025; Qin et al., 2012; Yang, 2015) involving 681 students and aimed to evaluate the effects of music-based interventions on test anxiety among students. The results showed that, compared with control conditions, music-based interventions significantly reduced students' test anxiety levels (SMD = −1.21, 95% CI: −1.79 to −0.63, *P* < 0.0001), suggesting a large intervention effect in alleviating anxiety responses in examination-related contexts. Further sensitivity analysis showed that the direction of the pooled effect remained consistent after sequentially excluding each individual study. In addition, after adjustment for potential publication bias using the trim-and-fill method, the effect size remained statistically significant, indicating that the conclusions of this study were robust.

The findings of this study further extend previous evidence on the effects of music-based interventions in improving anxiety symptoms. Previous meta-analyses have primarily focused on general anxiety or state anxiety and have shown that music-based interventions and music listening can reduce anxiety levels (de Witte et al., 2025; Harney et al., 2023; Li et al., 2026; Chen et al., 2026). However, general anxiety and test anxiety differ in their eliciting factors and psychological processes. Test anxiety is highly context-specific and typically occurs in situations involving examination evaluation, performance feedback, and academic competition. Its core manifestations include not only tension and physiological arousal but also worry about failure, negative self-evaluation, and impaired attentional resources (Tan et al., 2025). Therefore, by evaluating test anxiety as an independent outcome, the present study further confirms the effectiveness of music-based interventions in specific academic stress contexts.

The intervention effects observed in this study may be attributable to the combined influence of music on physiological arousal, emotional regulation, and cognitive processing.

First, at the physiological level, musical stimulation can modulate autonomic nervous system activity, enhance parasympathetic activation, and reduce the excessive physiological arousal associated with anxiety states (Liu et al., 2026). Previous studies have shown that listening to slow-tempo music with soothing melodies can activate the parasympathetic nervous system, reduce heart rate and blood pressure, and decrease cortisol secretion (Darki et al., 2022), thereby directly counteracting anxiety-related physiological hyperarousal. This process may occur through modulation of the hypothalamic-pituitary-adrenal axis and autonomic regulatory centers, shifting the body from a “fight-or-flight” state toward relaxation and recovery (McPherson et al., 2019). Students with test anxiety often experience pronounced tension responses and somatic symptoms, and music-based interventions may alleviate pre-examination physiological tension by reducing sympathetic nervous system activity, thereby improving the anxiety experience.

Second, at the psychological level, music may exert its effects through attentional and emotional regulation mechanisms. Students with test anxiety are often prone to catastrophic thinking, such as excessive concern about examination failure, declining grades, and future uncertainty, and these negative cognitions may further increase anxiety levels (Mojarrab, 2020). As an attention-capturing stimulus, music may help individuals temporarily disengage from stress-related thoughts and redirect attentional resources from anxiety-related cognitions to the musical experience, thereby reducing the cognitive load involved in the maintenance of anxiety (Kiss and Linnell, 2024; Symons and Tierney, 2024). In addition, familiar music or music with positive emotional significance may activate positive emotional experiences, enhance psychological safety and emotional stability, and help strengthen students' psychological regulation capacity when facing examination-related stress (Ren et al., 2024; Talamini et al., 2022).

At the neurobiological level, musical stimulation may influence activity in brain regions involved in emotional processing and stress regulation. Previous studies have shown that music can modulate functional activity in regions such as the amygdala, pre-frontal cortex, and limbic system, thereby reducing emotional responses to threatening stimuli and facilitating cognitive reappraisal processes (Fuentes-Sánchez et al., 2025; Xu and Li, 2025). For students with test anxiety, music may help them cope more effectively with examination-related stress by reducing the intensity of emotional responses (Trevor, 2024) and enhancing cognitive control capacity (Sayal et al., 2025).

Overall, the findings of this study suggest that music-based interventions do not reduce anxiety merely through a transient recreational experience; rather, they may exert their effects through multiple pathways, including physiological relaxation, emotional regulation, and cognitive control. This may also explain why positive effects of music-based interventions were observed among students across different educational stages.

### 4.2 Interpretation of subgroup analysis results and influencing factors

To further explore potential sources of variation in the effects of music-based interventions, subgroup analyses were conducted according to intervention duration, session duration, intervention frequency, intervention modality, and target population. The results indicated that different intervention parameters may influence the effectiveness of music-based interventions in reducing test anxiety.

#### 4.2.1 Effects of intervention duration and session duration

This study found that longer music-based intervention periods were associated with greater improvements in test anxiety. In particular, the subgroup with an intervention duration of ≥8 weeks showed the largest effect size (SMD = −2.14), which was substantially greater than that observed for interventions lasting < 4 weeks (SMD = −0.43). This finding suggests that music-based interventions may require a certain period of accumulation to produce stable psychological regulation effects. It is also consistent with theories of habit formation and the development of psychological regulation capacity (Ito et al., 2024; Rossi et al., 2024). Improvement in test anxiety may depend not only on immediate emotional relief but also on the gradual development of new stress-coping strategies. Long-term and regular music activities may help students establish a stable association between music, relaxation experiences, and emotional regulation, enabling music to gradually become a regulatory strategy that can be actively used when facing examination-related stress (Luo et al., 2025; Taipale et al., 2025). Therefore, when implementing music-based intervention programs, schools should not rely solely on short-term intensive interventions before examinations but should consider incorporating them into ongoing mental health education activities.

The present study also found that sessions lasting ≥60 min were more effective than shorter sessions (SMD = −1.89). This finding may be related to the time required for the musical experience to reach a sufficient state of emotional regulation. Music-based interventions typically involve several stages, including attentional engagement, emotional involvement, and physical relaxation. Shorter exposure to music may produce only immediate emotional improvement, whereas sufficient intervention time may be more conducive to establishing a stable relaxation response (Chee et al., 2024; Jia et al., 2016). However, it should be noted that the effects of music-based interventions do not simply depend on extending session duration. Excessively long music exposure may reduce students' active engagement and may even interfere with the completion of learning tasks. Therefore, in school-based applications, the intervention dose should be reasonably designed according to students' age characteristics and practical academic schedules.

#### 4.2.2 Effects of intervention modality

The subgroup analysis showed that active music-based interventions (SMD = −2.21) produced a larger effect size than receptive music-based interventions (SMD = −0.50), suggesting that the degree of musical engagement may be an important factor influencing intervention effectiveness.

Receptive music-based interventions mainly rely on the relaxation effects induced by music listening and may alleviate anxiety by reducing physiological arousal and improving emotional states. In contrast, active music-based interventions, such as songwriting, instrumental performance, and improvisational expression, involve not only musical stimulation but also active participation, emotional expression, and social interaction. Therefore, they may produce more complex psychological regulation effects (Schneider et al., 2022).

Active music-based interventions may enhance students' self-efficacy and sense of control, both of which are important psychological factors in reducing test anxiety. Students with test anxiety often worry about the uncontrollability of examination outcomes, whereas active music activities can provide opportunities for goal setting, feedback, and successful experiences, thereby improving students' cognitive appraisal of stressful events (Stekic, 2024). At the same time, active music-based interventions usually require guidance from trained professionals as well as appropriate venues and equipment.

Therefore, when implementing these interventions in school settings, the choice of intervention modality should be based on available resources. For large-scale implementation, simple and easy-to-deliver receptive music-based interventions may be adopted. For students with more severe test anxiety or those requiring more intensive psychological support, group-based active music-based intervention activities may be considered.

#### 4.2.3 Effects of the target population on intervention outcomes

The subgroup analysis showed that music-based interventions produced a more pronounced improvement in test anxiety among secondary school students (SMD = −2.14), whereas the effect size was relatively smaller among university students (SMD = −0.75). This finding suggests that students' developmental stage and examination-related stress context may be important factors influencing the effectiveness of music-based interventions. Previous research supports this interpretation. Compared with routine course examinations among university students, secondary school students usually face greater pressure from high-stakes selection examinations, such as senior high school entrance examinations and college entrance examinations, and may therefore have higher baseline anxiety levels and greater room for improvement after intervention (Dutt et al., 2025).

### 4.3 Applicability and practical implications of music-based interventions as adjunctive measures in school-based mental health education

The findings of this study indicate that music-based interventions can significantly reduce students' test anxiety levels, with positive effects observed across different intervention durations, intervention modalities, and student populations. These findings suggest that music-based interventions may have potential value as adjunctive measures in school-based mental health education (Sagirkaya and Torun, 2026; Ugwuanyi et al., 2020; Inangil et al., 2020; Ergin et al., 2025; Contreras, 2022; Liang, 2024; Song et al., 2024, 2025; Qin et al., 2012; Yang, 2015).

Based on the subgroup analysis results and the characteristics of the included studies, the application of music-based interventions as adjunctive measures in school-based mental health education should meet several conditions: (1) music-based interventions may be more suitable for students with test anxiety; (2) their effectiveness depends on standardized intervention protocols rather than simple music playback. The subgroup analyses showed that interventions lasting ≥8 weeks, sessions lasting ≥60 min, and active music-based interventions may produce more pronounced reductions in anxiety; (3) music type and individual adaptation are important factors influencing intervention effects. The included studies used various types of music, including classical music, folk music, light music, and music selected according to individual preferences. Different musical characteristics may influence students' emotional experiences through rhythm, melody, familiarity, and cultural identification; and (4) the effectiveness of implementation is also influenced by school settings and available resources. Compared with psychotherapeutic approaches requiring long-term involvement of trained professionals, music-based interventions have advantages such as lower cost, greater flexibility, and higher student acceptability, making them particularly suitable for preventive and universal applications in school-based mental health education.

Therefore, the use of music-based interventions as adjunctive measures in school-based mental health education has clear boundaries of applicability. Specifically, they may be more appropriate for students with test anxiety in school settings and should be implemented with standardized design, appropriate music selection, and professional guidance. Thus, music-based interventions should not be regarded as a substitute for traditional mental health services but rather should be integrated with mental health courses, psychological counseling, and other evidence-based psychological interventions to establish a multilevel student mental health support system.

### 4.4 Strengths and limitations

This study has several strengths. First, it is among the first meta-analyses to specifically focus on “music-based interventions for test anxiety among students.” Compared with previous systematic reviews that addressed test anxiety only as a subtype or secondary dimension of anxiety, the present study has a more targeted research focus. Second, the literature search was comprehensive, covering eight Chinese and English databases, including CNKI, Wanfang Data, VIP Database, CBM, PubMed, Web of Science, the Cochrane Library, and Embase, with the search updated to April 2026, thereby ensuring the timeliness and comprehensiveness of the included evidence. Third, this study is the first to systematically conduct multidimensional subgroup analyses across four domains, namely intervention duration, session duration, intervention modality (active/receptive), and target population (secondary school/university students). These analyses helped identify potentially optimal intervention parameters for alleviating test anxiety through music-based interventions and provide direct evidence for stratified intervention strategies in school-based mental health services. Fourth, the trim-and-fill method was used to adjust for publication bias, and the direction of the adjusted pooled effect size was consistent with that of the original analysis, supporting the robustness of the conclusions.

This study conducted a comprehensive literature search and included a broad range of eligible studies. Nevertheless, several limitations should be considered when interpreting the findings: (1) owing to the inherent nature of music-based interventions, blinding of participants and intervention providers was not feasible in any included study. In addition, randomization procedures were insufficiently reported in some studies, resulting in moderate overall methodological quality; (2) differences in intervention protocols, control conditions, and outcome measurement instruments contributed to substantial statistical heterogeneity. Although subgroup analyses were conducted, some sources of heterogeneity could not be fully explained; (3) the included studies were geographically concentrated, primarily in China and Türkiye, and the generalizability of the findings requires further validation; (4) most studies did not report follow-up data, precluding assessment of the long-term effects of music-based interventions; and (5) a large number of records were excluded because full texts were unavailable or because studies relied on self-report scales. Despite these limitations, the findings provide valuable evidence to inform school-based mental health education.

## 5 Conclusions

This systematic review and meta-analysis demonstrated that music-based interventions significantly reduce test anxiety among students and may serve as an effective adjunct to school-based mental health education and test anxiety management. Future research should conduct additional high-quality, large-sample, multicenter randomized controlled trials using rigorously designed and comprehensively reported music-based intervention protocols. Further studies should also compare different types of music and intervention doses and incorporate long-term follow-up and neurobiological investigations to provide more robust evidence for developing standardized intervention protocols and evaluating both the short- and long-term effects of music-based interventions.

## Data Availability

The original contributions presented in the study are included in the article/Supplementary material, further inquiries can be directed to the corresponding author.
