# Supplementary material for: Effects of music-based interventions on test anxiety in students: a systematic review and meta-analysis
Source: Front Psychol. 2026 Aug 20;17:1922862. doi: 10.3389/fpsyg.2026.1922862 (PMC13538006; doi:10.3389/fpsyg.2026.1922862)
Supplement: Supplementary file 1 [file Table_1.docx]

| **Table S1** The search strategy | | |
| --- | --- | --- |
| **Datebases** | **Searching strategy** | **Literature number** |
| Pubmed | (((((((((((((((((((((((((((((((Music therapy[MeSH Terms]) OR (Music[MeSH Terms])) OR (Music therapy[Title/Abstract])) OR (Music[Title/Abstract])) OR (Therapy, Music[Title/Abstract])) OR (music intervention[Title/Abstract])) OR (Music Medicine[Title/Abstract])) OR (Receptive music therapy[Title/Abstract])) OR (musical instrument performance[Title/Abstract])) OR (Music listening[Title/Abstract])) OR (Five Elements Music[Title/Abstract])) OR (Singing[Title/Abstract])) OR (Instrument Playing[Title/Abstract])) OR (Active music therapy[Title/Abstract])) OR (Songs[Title/Abstract])) OR (Song[Title/Abstract])) OR (Vocal Melody[Title/Abstract])) OR (Melodies, Vocal[Title/Abstract])) OR (Melody, Vocal[Title/Abstract])) OR (Vocal Melodies[Title/Abstract])) OR (Classical Music[Title/Abstract])) OR (Music, Classical[Title/Abstract])) OR (Jazz Music[Title/Abstract])) OR (Music, Jazz[Title/Abstract])) OR (Rap Music[Title/Abstract])) OR (Music, Rap[Title/Abstract])) OR (Hip Hop Music[Title/Abstract])) OR (Hop Music, Hip[Title/Abstract])) OR (Music, Hip Hop[Title/Abstract])) OR (Rock[Title/Abstract] AND Roll Music[Title/Abstract])) AND (((((((((((((((((((((((((Students[MeSH Terms]) OR (Adolescent[MeSH Terms])) OR (Students[Title/Abstract])) OR (Adolescent[Title/Abstract])) OR (Adolescents[Title/Abstract])) OR (Adolescence[Title/Abstract])) OR (Adolescents, Female[Title/Abstract])) OR (Adolescent, Female[Title/Abstract])) OR (Female Adolescent[Title/Abstract])) OR (Female Adolescents[Title/Abstract])) OR (Adolescents, Male[Title/Abstract])) OR (Adolescent, Male[Title/Abstract])) OR (Male Adolescent[Title/Abstract])) OR (Male Adolescents[Title/Abstract])) OR (Youth[Title/Abstract])) OR (Youths[Title/Abstract])) OR (Teens[Title/Abstract])) OR (Teen[Title/Abstract])) OR (Teenagers[Title/Abstract])) OR (Teenager[Title/Abstract])) OR (Student[Title/Abstract])) OR (School Enrollment[Title/Abstract])) OR (Enrollment, School[Title/Abstract])) OR (Enrollments, School[Title/Abstract])) OR (School Enrollments[Title/Abstract]))) AND (((((((((((((((((((((((((((((((((Anxiety[MeSH Terms]) OR (Anxiety Disorders[MeSH Terms])) OR (Test Anxiety[MeSH Terms])) OR (Anxiety[Title/Abstract])) OR (Anxiety Disorders[Title/Abstract])) OR (Test Anxiety[Title/Abstract])) OR (Angst[Title/Abstract])) OR (Nervousness[Title/Abstract])) OR (Hypervigilance[Title/Abstract])) OR (Social Anxiety[Title/Abstract])) OR (Anxieties, Social[Title/Abstract])) OR (Anxiety, Social[Title/Abstract])) OR (Social Anxieties[Title/Abstract])) OR (Anxiousness[Title/Abstract])) OR (Anxiety Disorder[Title/Abstract])) OR (Disorder, Anxiety[Title/Abstract])) OR (Disorders, Anxiety[Title/Abstract])) OR (Neuroses, Anxiety[Title/Abstract])) OR (Anxiety Neuroses[Title/Abstract])) OR (Anxiety States, Neurotic[Title/Abstract])) OR (Anxiety State, Neurotic[Title/Abstract])) OR (Neurotic Anxiety State[Title/Abstract])) OR (Neurotic Anxiety States[Title/Abstract])) OR (State, Neurotic Anxiety[Title/Abstract])) OR (States, Neurotic Anxiety[Title/Abstract])) OR (Anxiety, Test[Title/Abstract])) OR (Pre-Exam Anxiety[Title/Abstract])) OR (Anxiety, Pre-Exam[Title/Abstract])) OR (Pre Exam Anxiety[Title/Abstract])) OR (Exam Anxiety[Title/Abstract])) OR (Anxiety, Exam[Title/Abstract])) OR (Exam Stress[Title/Abstract])) OR (Stress, Exam[Title/Abstract])) | **651** |
| Web of science | ( TS=("Music therapy") OR TS=("Music") OR TS=("Therapy, Music") OR TS=("music intervention") OR TS=("Music Medicine") OR TS=("Receptive music therapy") OR TS=("musical instrument performance") OR TS=("Music listening") OR TS=("Five Elements Music") OR TS=("Singing") OR TS=("Instrument Playing") OR TS=("Active music therapy") OR TS=("Songs") OR TS=("Song") OR TS=("Vocal Melody") OR TS=("Melodies, Vocal") OR TS=("Melody, Vocal") OR TS=("Vocal Melodies") OR TS=("Classical Music") OR TS=("Music, Classical") OR TS=("Jazz Music") OR TS=("Music, Jazz") OR TS=("Rap Music") OR TS=("Music, Rap") OR TS=("Hip Hop Music") OR TS=("Hop Music, Hip") OR TS=("Music, Hip Hop") OR TS=(Rock AND "Roll Music") )  AND  ( TS=("Students") OR TS=("Adolescent") OR TS=("Adolescents") OR TS=("Adolescence") OR TS=("Adolescents, Female") OR TS=("Adolescent, Female") OR TS=("Female Adolescent") OR TS=("Female Adolescents") OR TS=("Adolescents, Male") OR TS=("Adolescent, Male") OR TS=("Male Adolescent") OR TS=("Male Adolescents") OR TS=("Youth") OR TS=("Youths") OR TS=("Teens") OR TS=("Teen") OR TS=("Teenagers") OR TS=("Teenager") OR TS=("Student") OR TS=("School Enrollment") OR TS=("Enrollment, School") OR TS=("Enrollments, School") OR TS=("School Enrollments") )  AND  ( TS=("Anxiety") OR TS=("Anxiety Disorders") OR TS=("Test Anxiety") OR TS=("Angst") OR TS=("Nervousness") OR TS=("Hypervigilance") OR TS=("Social Anxiety") OR TS=("Anxieties, Social") OR TS=("Anxiety, Social") OR TS=("Social Anxieties") OR TS=("Anxiousness") OR TS=("Anxiety Disorder") OR TS=("Disorder, Anxiety") OR TS=("Disorders, Anxiety") OR TS=("Neuroses, Anxiety") OR TS=("Anxiety Neuroses") OR TS=("Anxiety States, Neurotic") OR TS=("Anxiety State, Neurotic") OR TS=("Neurotic Anxiety State") OR TS=("Neurotic Anxiety States") OR TS=("State, Neurotic Anxiety") OR TS=("States, Neurotic Anxiety") OR TS=("Anxiety, Test") OR TS=("Pre-Exam Anxiety") OR TS=("Anxiety, Pre-Exam") OR TS=("Pre Exam Anxiety") OR TS=("Exam Anxiety") OR TS=("Anxiety, Exam") OR TS=("Exam Stress") OR TS=("Stress, Exam") ) | **820** |
| Embase | Query \| Results; #31 AND #57 AND #91 \| 1144; #58 OR #59 OR #60 OR #61 OR #62 OR #63 OR #64 OR #65 OR #66 OR #67 OR #68 OR #69 OR #70 OR #71 OR #72 OR #73 OR #74 OR #75 OR #76 OR #77 OR #78 OR #79 OR #80 OR #81 OR #82 OR #83 OR #84 OR #85 OR #86 OR #87 OR #88 OR #89 OR #90 \| 844043; 'stress, exam':ab,ti \| 29; 'exam stress':ab,ti \| 164; 'anxiety, exam':ab,ti \| 3; 'exam anxiety':ab,ti \| 146; 'pre exam anxiety':ab,ti \| 23; 'anxiety, pre-exam':ab,ti \| 1; 'pre-exam anxiety':ab,ti \| 23; 'anxiety, test':ab,ti \| 512; 'states, neurotic anxiety':ab,ti \| 0; 'state, neurotic anxiety':ab,ti \| 0; 'neurotic anxiety states':ab,ti \| 3; 'neurotic anxiety state':ab,ti \| 1; 'anxiety state, neurotic':ab,ti \| 0; 'anxiety states, neurotic':ab,ti \| 0; 'anxiety neuroses':ab,ti \| 90; 'neuroses, anxiety':ab,ti \| 2; 'disorders, anxiety':ab,ti \| 2525; 'disorder, anxiety':ab,ti \| 1964; 'anxiety disorder':ab,ti \| 38264; 'anxiousness':ab,ti \| 665; 'social anxieties':ab,ti \| 67; 'anxiety, social':ab,ti \| 1413; 'anxieties, social':ab,ti \| 2; 'social anxiety':ab,ti \| 12483; 'hypervigilance':ab,ti \| 2023; 'nervousness':ab,ti \| 3602; 'angst':ab,ti \| 610; 'test anxiety':ab,ti \| 1648; 'anxiety disorders':ab,ti \| 39833; 'anxiety':ab,ti \| 487656; 'test anxiety'/exp \| 401; 'anxiety disorders'/exp \| 416418; 'anxiety'/exp \| 412305; #32 OR #33 OR #34 OR #35 OR #36 OR #37 OR #38 OR #39 OR #40 OR #41 OR #42 OR #43 OR #44 OR #45 OR #46 OR #47 OR #48 OR #49 OR #50 OR #51 OR #52 OR #53 OR #54 OR #55 OR #56 \| 2916106; 'school enrollments':ab,ti \| 52; 'enrollments, school':ab,ti \| 0; 'enrollment, school':ab,ti \| 5; 'school enrollment':ab,ti \| 638; 'student':ab,ti \| 251349; 'teenager':ab,ti \| 5903; 'teenagers':ab,ti \| 23156; 'teen':ab,ti \| 10058; 'teens':ab,ti \| 12838; 'youths':ab,ti \| 21236; 'youth':ab,ti \| 139198; 'male adolescents':ab,ti \| 4177; 'male adolescent':ab,ti \| 1712; 'adolescent, male':ab,ti \| 3392; 'adolescents, male':ab,ti \| 169; 'female adolescents':ab,ti \| 7091; 'female adolescent':ab,ti \| 2228; 'adolescent, female':ab,ti \| 3017; 'adolescents, female':ab,ti \| 106; 'adolescence':ab,ti \| 107125; 'adolescentst':ab,ti \| 2; 'adolescent':ab,ti \| 240788; 'students':ab,ti \| 483985; 'adolescent'/exp \| 2224320; 'students'/exp \| 391905; #1 OR #2 OR #3 OR #4 OR #5 OR #6 OR #7 OR #8 OR #9 OR #10 OR #11 OR #12 OR #13 OR #14 OR #15 OR #16 OR #17 OR #18 OR #19 OR #20 OR #21 OR #22 OR #23 OR #24 OR #25 OR #26 OR #27 OR #28 OR #29 OR #30 \| 61002; 'rock and roll music':ab,ti \| 19; 'music, hip hop':ab,ti \| 1; 'hop music, hip':ab,ti \| 0; 'hip hop music':ab,ti \| 36; 'music, rap':ab,ti \| 8; 'rap music':ab,ti \| 92; 'music, jazz':ab,ti \| 8; 'jazz music':ab,ti \| 39; 'music, classical':ab,ti \| 54; 'classical music':ab,ti \| 862; 'vocal melodies':ab,ti \| 12; 'melody, vocal':ab,ti \| 3 | **1144** |
| Cochrane Library | #1 (Music therapy):ti,ab,kw OR (Music):ti,ab,kw OR (Therapy, Music):ti,ab,kw OR (music intervention):ti,ab,kw OR (Music Medicine):ti,ab,kw 9248  #2 (Receptive music therapy):ti,ab,kw OR (musical instrument performance):ti,ab,kw OR (Music listening):ti,ab,kw OR (Five Elements Music):ti,ab,kw OR (Singing):ti,ab,kw 2727  #3 (Instrument Playing):ti,ab,kw OR (Active music therapy):ti,ab,kw OR (Songs):ti,ab,kw OR (Song):ti,ab,kw OR (Vocal Melody):ti,ab,kw 1303  #4 (Melodies, Vocal):ti,ab,kw OR (Melody, Vocal):ti,ab,kw OR (Vocal Melodies):ti,ab,kw OR (Classical Music):ti,ab,kw OR (Music, Classical):ti,ab,kw 486  #5 (Jazz Music):ti,ab,kw OR (Music, Jazz):ti,ab,kw OR (Rap Music):ti,ab,kw OR (Music, Rap):ti,ab,kw OR (Hip Hop Music):ti,ab,kw 74  #6 (Hop Music, Hip):ti,ab,kw OR (Music, Hip Hop):ti,ab,kw OR (Rock and Roll Music):ti,ab,kw 17  #7 #1 OR #2 OR #3 OR #4 OR #5 OR #6 10053  #8 (Students):ti,ab,kw OR (Adolescent):ti,ab,kw OR (Adolescents):ti,ab,kw OR (Adolescence):ti,ab,kw OR (Adolescents, Female):ti,ab,kw 214352  #9 (Adolescent, Female):ti,ab,kw OR (Female Adolescent):ti,ab,kw OR (Female Adolescents):ti,ab,kw OR (Adolescents, Male):ti,ab,kw OR (Adolescent, Male):ti,ab,kw 149067  #10 (Male Adolescent):ti,ab,kw OR (Male Adolescents):ti,ab,kw OR (Youth):ti,ab,kw OR (Youths):ti,ab,kw OR (Teens):ti,ab,kw 140115  #11 (Teen):ti,ab,kw OR (Teenagers):ti,ab,kw OR (Teenager):ti,ab,kw OR (Student):ti,ab,kw OR (School Enrollment):ti,ab,kw 37113  #12 (Enrollment, School):ti,ab,kw OR (Enrollments, School):ti,ab,kw OR (School Enrollments):ti,ab,kw 1502  #13 #8 OR #9 OR #10 OR #11 OR #12 235072  #14 (Anxiety):ti,ab,kw OR (Anxiety Disorders):ti,ab,kw OR (Test Anxiety):ti,ab,kw OR (Angst):ti,ab,kw OR (Nervousness):ti,ab,kw 95819  #15 (Hypervigilance):ti,ab,kw OR (Social Anxiety):ti,ab,kw OR (Anxieties, Social):ti,ab,kw OR (Anxiety, Social):ti,ab,kw OR (Social Anxieties):ti,ab,kw 15172  #16 (Anxiousness):ti,ab,kw OR (Anxiety Disorder):ti,ab,kw OR (Disorder, Anxiety):ti,ab,kw OR (Disorders, Anxiety):ti,ab,kw OR (Neuroses, Anxiety):ti,ab,kw 40346  #17 (Anxiety Neuroses):ti,ab,kw OR (Anxiety States, Neurotic):ti,ab,kw OR (Anxiety State, Neurotic):ti,ab,kw OR (Neurotic Anxiety State):ti,ab,kw OR (Neurotic Anxiety States):ti,ab,kw 104  #18 (State, Neurotic Anxiety):ti,ab,kw OR (States, Neurotic Anxiety):ti,ab,kw OR (Anxiety, Test):ti,ab,kw OR (Pre-Exam Anxiety):ti,ab,kw OR (Anxiety, Pre-Exam):ti,ab,kw 20070  #19 (Pre Exam Anxiety):ti,ab,kw OR (Exam Anxiety):ti,ab,kw OR (Anxiety, Exam):ti,ab,kw OR (Exam Stress):ti,ab,kw OR (Stress, Exam):ti,ab,kw 827  #20 #14 OR #15 OR #16 OR #17 OR #18 OR #19 96261  #21 #7 AND #13 AND #20 495 | **491** |
| CNKI | TKA=('音乐治疗'+'音乐疗法'+'音乐干预'+'五行音乐'+'五音疗法'+'接受式音乐治疗'+'音乐引导想象'+'歌唱'+'器乐演奏'+'音乐放松') AND TKA=('学生'+'中学生'+'初中生'+'高中生'+'青少年'+'青年'+'少年'+'青春期'+'年轻人'+'未成年人'+'中学学生'+'低龄青少年'+'大龄青少年'+'在校学生'+'中学生群体'+'高中生群体'+'青少年学生'+'学龄青少年'+'中学生人群'+'未成年学生'+'青少年人群'+'初高中学生'+'青少年群体') AND TKA=('焦虑'+'焦虑情绪'+'焦虑症状'+'焦虑状态'+'焦虑障碍'+'紧张情绪'+'心理焦虑'+'考试焦虑'+'学业焦虑'+'测试焦虑') | **225** |
| Wanfang Data | 主题:("音乐治疗" OR "音乐疗法" OR "音乐干预" OR "五行音乐" OR "五音疗法" OR "接受式音乐治疗" OR "音乐引导想象" OR "歌唱" OR "器乐演奏" OR "音乐放松") AND 主题:("学生" OR "中学生" OR "初中生" OR "高中生" OR "青少年" OR "青年" OR "少年" OR "青春期" OR "年轻人" OR "未成年人" OR "中学学生" OR "低龄青少年" OR "大龄青少年" OR "在校学生" OR "中学生群体" OR "高中生群体" OR "青少年学生" OR "学龄青少年" OR "中学生人群" OR "未成年学生" OR "青少年人群" OR "初高中学生" OR "中学生群体" OR "青少年群体") AND 主题:("焦虑" OR "焦虑情绪" OR "焦虑症状" OR "焦虑状态" OR "焦虑障碍" OR "紧张情绪" OR "心理焦虑" OR "考试焦虑" OR "学业焦虑" OR "测试焦虑") | **325** |
| CBM | (音乐治疗 OR 音乐疗法 OR 音乐干预 OR 五行音乐 OR 五音疗法 OR 接受式音乐治疗 OR 音乐引导想象 OR 歌唱 OR 器乐演奏 OR 音乐放松) AND (学生 OR 高校学生 OR 研究生 OR 专科生 OR 本科生 OR 医学生 OR 中学生 OR 初中生 OR 高中生 OR 青少年 OR 少年 OR 青春期 OR 未成年人 OR 青少年学生 OR 学龄青少年 OR 初高中学生) AND (焦虑 OR 焦虑情绪 OR 焦虑症状 OR 焦虑状态 OR 焦虑障碍 OR 紧张情绪 OR 心理焦虑 OR 考试焦虑 OR 学业焦虑 OR 测试焦虑) | **3** |
| VIP Database | M=("音乐治疗" OR "音乐疗法" OR "音乐干预" OR "五行音乐" OR "五音疗法" OR "音乐" OR "接受式音乐治疗" OR "音乐引导想象" OR "歌唱" OR "器乐演奏" OR "音乐放松") AND M=("学生" OR "中学生" OR "初中生" OR "高中生" OR "青少年" OR "青年" OR "少年" OR "青春期" OR "年轻人" OR "未成年人" OR "中学学生" OR "低龄青少年" OR "大龄青少年" OR "在校学生" OR "中学生群体" OR "高中生群体" OR "青少年学生" OR "学龄青少年" OR "中学生人群" OR "未成年学生" OR "青少年人群" OR "初高中学生" OR "中学生群体" OR "青少年群体") AND M=("焦虑" OR "焦虑情绪" OR "焦虑症状" OR "焦虑状态" OR "焦虑障碍" OR "紧张情绪" OR "心理焦虑" OR "考试焦虑" OR "学业焦虑" OR "测试焦虑") | **119** |
